# Heterologous over-expression of *ACC SYNTHASE8 (ACS8)* in *Populus tremula x P. alba* clone 717-1B4 results in elevated levels of ethylene and induces stem dwarfism and reduced leaf size through separate genetic pathways

**DOI:** 10.3389/fpls.2014.00514

**Published:** 2014-11-04

**Authors:** Jonathan M. Plett, Martin Williams, Gaetan LeClair, Sharon Regan, Tannis Beardmore

**Affiliations:** ^1^Department of Biology, Queen's UniversityKingston, ON, Canada; ^2^Hawkesbury Institute for the Environment, University of Western SydneyRichmond, NSW, Australia; ^3^Atlantic Forestry Centre, Canadian Forest Service, Natural Resources CanadaFredericton, NB, Canada

**Keywords:** poplar, activation tagging, wood formation, plant stature, plant growth rate

## Abstract

Plant height is an important agronomic and horticultural trait that impacts plant productivity, durability and esthetic appeal. A number of the plant hormones such as gibberellic acid (GA), auxin and ethylene have been linked to control of plant architecture and size. Reduction in GA synthesis and auxin transport result in dwarfism while ethylene may have a permissive or repressive effect on tissue growth depending upon the age of plant tissues or the environmental conditions considered. We describe here an activation-tagged mutant of *Populus tremula x P. alba* clone 717-1B4 identified from 2000 independent transgenic lines due to its significantly reduced growth rate and smaller leaf size. Named *dwarfy*, the phenotype is due to increased expression of *PtaACC SYNTHASE8*, which codes for an enzyme in the first committed step in the biosynthesis of ethylene. Stems of *dwarfy* contain fiber and vessel elements that are reduced in length while leaves contain fewer cells. These morphological differences are linked to *PtaACS8* inducing different transcriptomic programs in the stem and leaf, with genes related to auxin diffusion and sensing being repressed in the stem and genes related to cell division found to be repressed in the leaves. Altogether, our study gives mechanistic insight into the genetics underpinning ethylene-induced dwarfism in a perennial model organism.

## Introduction

Reduced plant height, or dwarfism, is an important agronomic trait linked to higher yields (Huang et al., 1996; Yang and Hwa, 2008), easier harvesting (Adkins et al., 2010) and reduced nutrient demand on soils (Sieling and Kage, 2008). Leaf size, meanwhile, is linked to productivity, predation (Faeth, 1991) and the water status of the plant (Scoffoni et al., 2011). While both height and leaf size are complex traits, they appear to be genetically regulated by a similar panel of plant hormones (Valdovinos et al., 1967; Ephritikhine et al., 1999; Qi et al., 2011; Luo et al., 2013) and cytochrome P450s (Zhang et al., 2014), as well as abiotic factors such as temperature (Yang et al., 2014) and photoperiod (Li et al., 2014). Reductions in organ size are a result of two different physiological phenomena: smaller cells and impeded cellular division (Beemster et al., 2003). These two factors may work independently or synergistically to affect plant stature and organ size (Beemster et al., 2005; Skirycz et al., 2010). Newly produced plant tissues first exhibit growth due to rapid cellular division, a phase that is replaced in a distal-proximal manner by cellular expansion in progenitor cells (Donnelly et al., 1999). Due to the integrated control between these two processes, genetic mutations to single genes can have a drastic impact on plant stature as a whole or at the level of a specific tissue. Altered expression of genes such as *ARABIDOPSIS VACUOLAR H+-PYROPHOSPHATASE1* (*AVP1*; Li et al., 2005), *CYTOKININ RESISTANT1* (*CNR1*; Guo et al., 2010), and *ISOPENTENYL TRANSFERASE3* (*IPT3*; Nobusawa et al., 2013) impact tissue size due to a difference in the total number of cells produced, while *EXPANSIN10* (*EXP10*; Cho and Cosgrove, 2000), *ARGOS-LIKE* (Hu et al., 2006), and *RETINOBLASTOMA-RELATED PROTEIN1* (*RBR1*; Sabelli et al., 2013) change the final size of plant tissues as a function of altered cell expansion.

The best studied genetic influences on dwarfism are genes and signaling pathways related to hormone production and sensitivity. Within these studies, ethylene, gibberellic acid (GA), auxin, and brassinosteroids (BR) have all been implicated with a role in cell division, cellular growth and overall plant architecture. Blocked BR synthesis (Nakaya et al., 2002) and reduced GA biosynthesis (Tong et al., 2007; Li et al., 2011) or increased GA catabolism (Busov et al., 2003; Schomburg et al., 2003; Curtis et al., 2005; Lee and Zeevaart, 2005; Dijkstra et al., 2008; Zawaski et al., 2011) induce dwarfism in a wide range of model plant systems. Auxin transport, meanwhile, is a critical component of proper plant stem elongation. In rice, auxin transport inhibition has been correlated to slower stem elongation (Yamamoto et al., 2007; Domingo et al., 2009) while reduced basipetal auxin transport in maize and *Arabidopsis thaliana* results in stunted plant development (Lantican and Muir, 1969; Geisler et al., 2003, 2005; Multani et al., 2003; Geisler and Murphy, 2006). Treatment of plant tissues with ethylene, a gaseous plant hormone, results in stunting (Vahala et al., 2013), a phenotype that has been linked to the induced expression of certain *ETHYLENE RESPONSE FACTORs* (*ERFs*; Dubois et al., 2013; Vahala et al., 2013). There also appears to be extensive cross-talk between the different hormone pathways with components of the ethylene pathway controlling GA biosynthesis (Qi et al., 2011) and the activity of DELLA proteins (Luo et al., 2013). Ethylene can also regulate auxin diffusion and biosynthesis (Valdovinos et al., 1967; Stepanova et al., 2005; Ruzicka et al., 2007; Swarup et al., 2007).

Here we characterize an activation tagged mutant of *Populus tremula x P. alba* clone 717, named “*dwarfy*,” exhibiting severe dwarfism with both reduced stature and smaller leaves. We show that the gene responsible for this phenotype is annotated as the poplar 1-aminocyclopropane-1-carboxylate synthase (ACS) gene *PtaACS8*. Ethylene is synthesized in two enzymatic steps from the substrate S-adenosyl-methionine (SAM). The first step is the conversion of SAM into 1-aminocyclopropane-1-carboxylic acid (ACC) by the activity of ACSs followed by the conversion of ACC to ethylene catalyzed by ACC OXIDASEs (ACOs). Ethylene is then perceived by a family of membrane bound receptors that induce the transcription of ETHYLENE RESPONSE FACTORs (ERFs) which, in turn, controls transcription and, ultimately, plant development. We demonstrate that increased expression of *PtaACS8* in the *dwarfy* line results in significantly higher levels of ethylene in all aerial tissues of the plant. Morphologically, the increased expression of *PtaACS8* in the stem results in shorter vessels and fibers in secondary growth while endogenous over-expression of the *PtaACS8* gene in the leaves results in the production of fewer cells. The reduced growth of stem cells is accompanied by a repression of auxin transport and signaling genes while reduction in cell number in leaves is concurrent with a large reduction in the transcript abundance of a number of cell-cycle genes. Therefore, we conclude that increased expression of *PtaACS8* induces stem dwarfism and reduced leaf size through separate genetic pathways.

## Materials and methods

### Plant material

**Figure 1:**
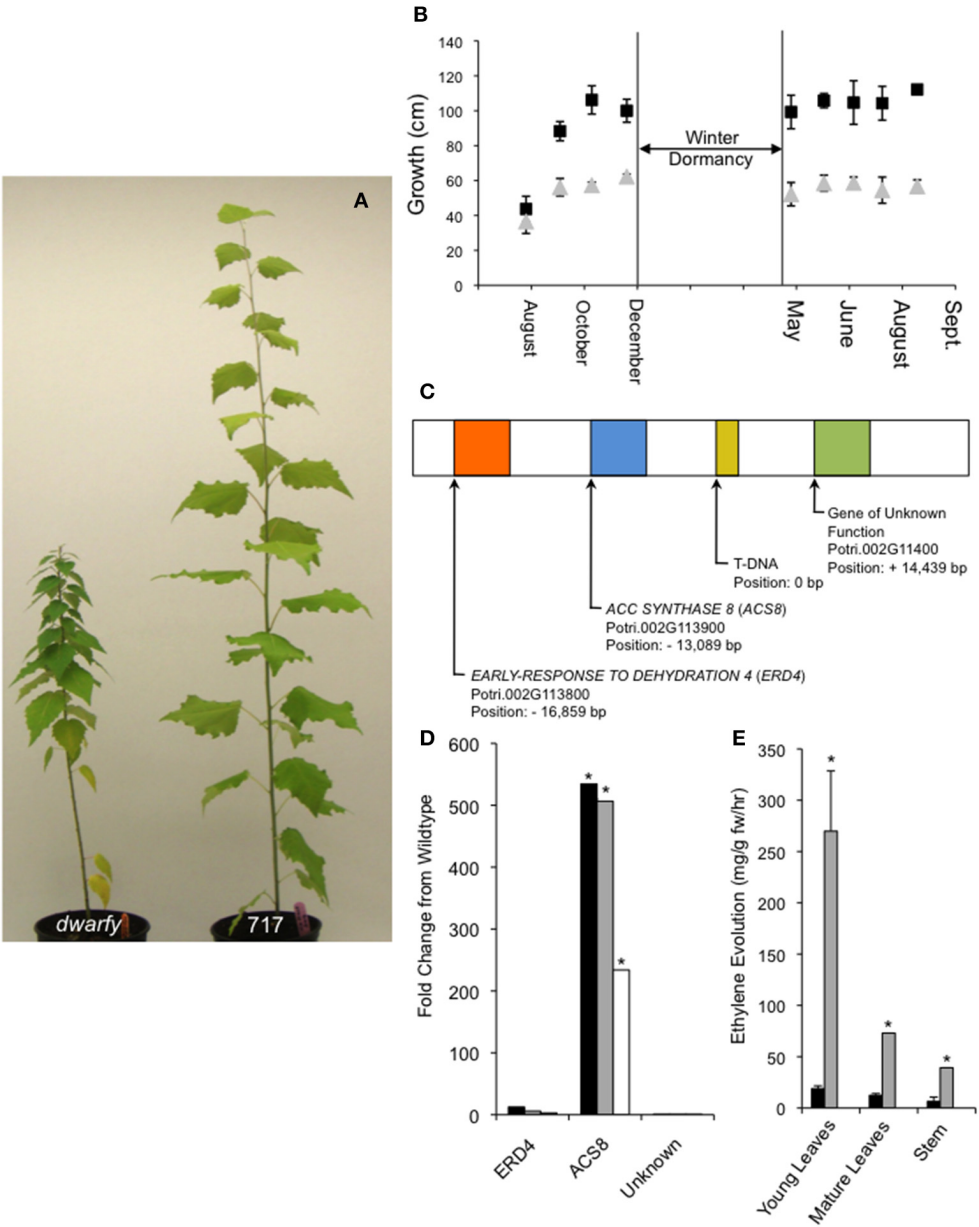
**Endogenous over-expression of *PtaACS8* results in a dwarfed growth phenotype in *P. tremula x P. alba* clone 717-1B4. (A)** A representative image of the *dwarfy* mutant *P. tremula x P. alba* clone as compared to wildtype (717) after 4 months of growth. **(B)** Graphical mean height growth of *dwarfy* (gray data points) and wildtype (black data points) over two growth seasons. **(C)** Graphical representation of the genes found within a 30 kb window around the insertion of the activation tagging T-DNA in the *dwarfy* mutant. **(D)** Fold change in the genes found in the genomic vicinity of the T-DNA insertion in the *dwarfy* mutant as compared to wildtype in immature leaves (black bars), mature leaves (gray bars), stem (white bars). **(E)** Comparison of ethylene evolution in three different tissues of *dwarfy* (gray bars) and wildtype (black bars). All values ±Standard Error (SE). In **(D,E)**, ^*^significantly different from wildtype (*p* < 0.05).

**Figure 2:**
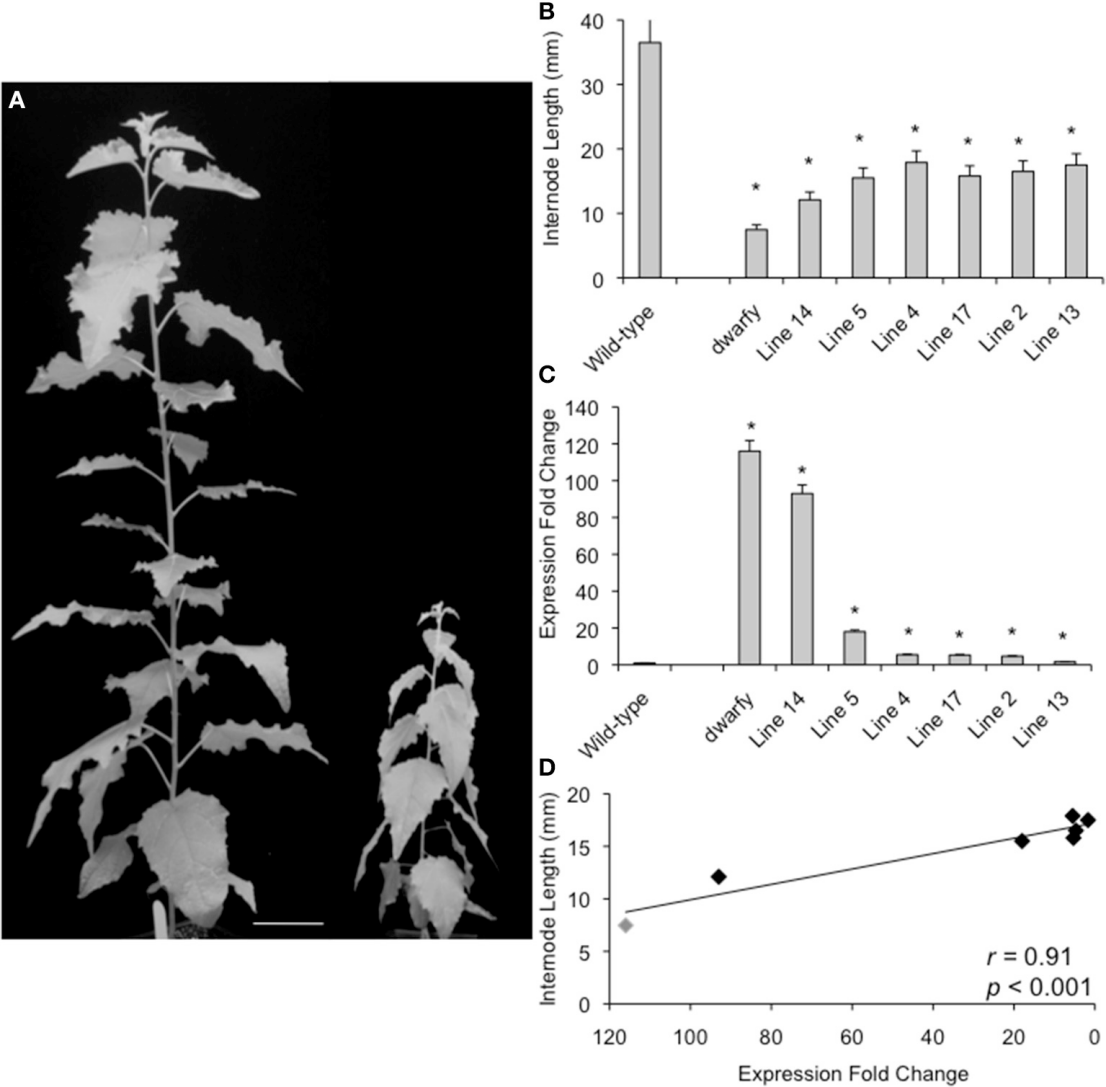
**Plant growth retardation in *dwarfy* mutant is significantly correlated to the expression level of *PtaACS8*. (A)** A representative image of one independent line of the *35S::PtaACS8* mutant *P. tremula x P. alba* clone as compared to wildtype (717) after 2 months of growth. Scale bar = 8 cm. **(B)** Relative expression of *PtaACS8* in wildtype, *dwarfy* and 6 independent transgenic lines containing the *35S::PtaACS8* construct. **(C)** Internode length of wildtype, *dwarfy* and 6 independent transgenic lines containing the *35S::PtaACS8* construct. **(D)** Correlation between *PtaACS8* expression levels and internode length in wildtype, *dwarfy* and 6 independent transgenic lines containing the *35S::PtaACS8* construct. All values ±Standard Error (SE). In **(B,C)**, ^*^significantly different from wildtype (*p* < 0.05).

**Figure 3:**
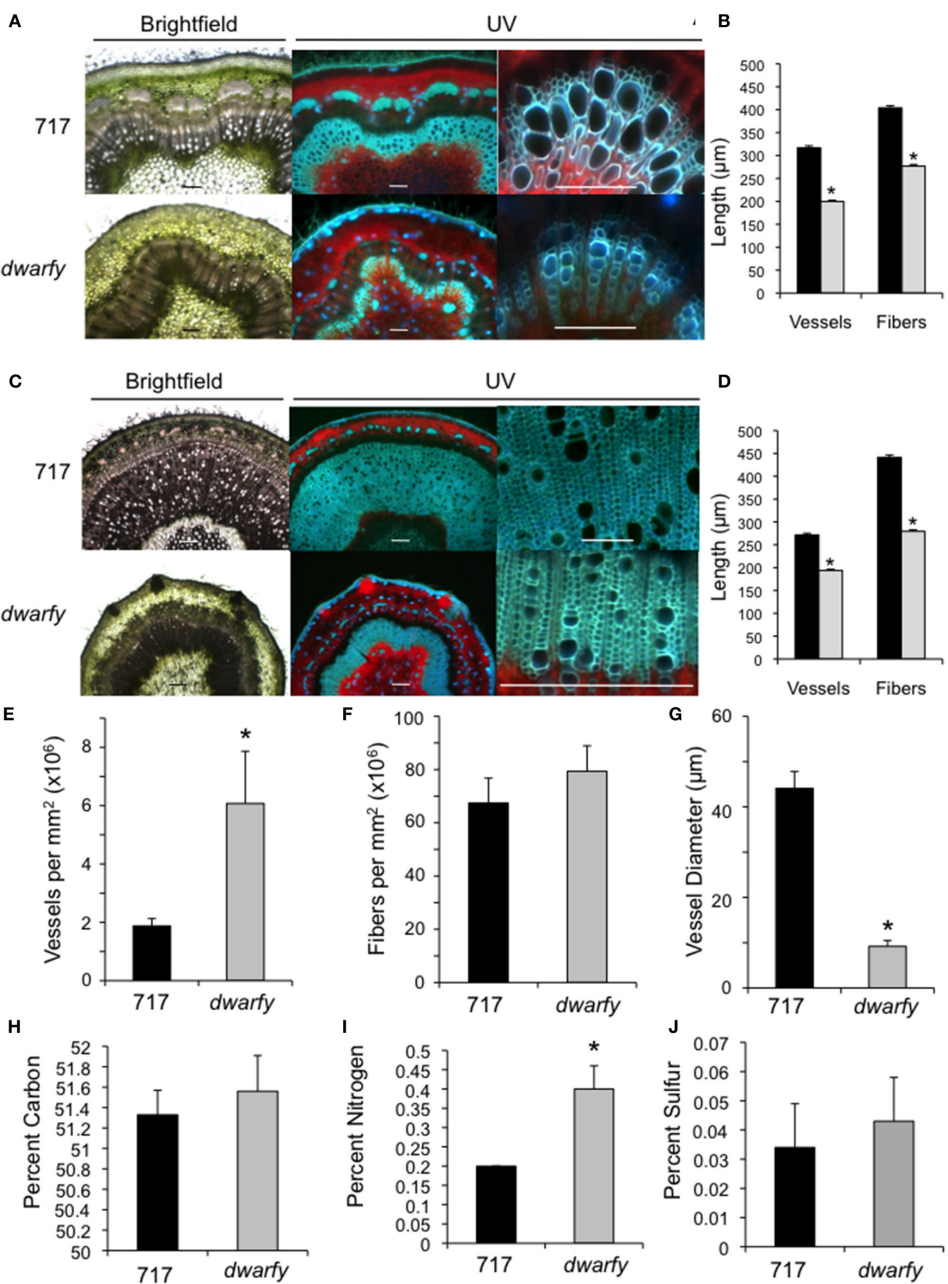
**Elevated expression levels of *PtaACS8* result in significant changes in stem architecture and physical characteristics. (A)** Transverse cross section of wild-type and *dwarfy* stems at the leaf 10–11 internode as observed under brightfield and UV autofluorescence. Scale bar = 1 mm for first two images and 100 μm for the third panel **(B)** Vessel and fiber lengths of wild-type (black bars) and *dwarfy* (gray bars) stems between the leaf 10–11 internode. **(C)** Transverse cross section of wildtype and *dwarfy* stems at the leaf 20–21 internode as observed under brightfield and UV autofluorescence. Scale bar = 0.5 mm for first two images and 100 μm for the third panel. **(D)** Vessel and fiber lengths of wild-type (black bars) and *dwarfy* (gray bars) stems between the leaf 20–21 internode. Vessel **(E)** and fiber **(F)** density in wild-type and *dwarfy* stems between the leaf 20–21 internode. **(G)** Vessel diameter in wild-type and *dwarfy* stems between the leaf 20–21 internode. Relative percentage of carbon **(H)**, nitrogen **(I)** and sulfur **(J)** in the stems of wildtype and *dwarfy*. All values ±Standard Error. ^*^Significantly different from wildtype (*p* < 0.05).

**Figure 4:**
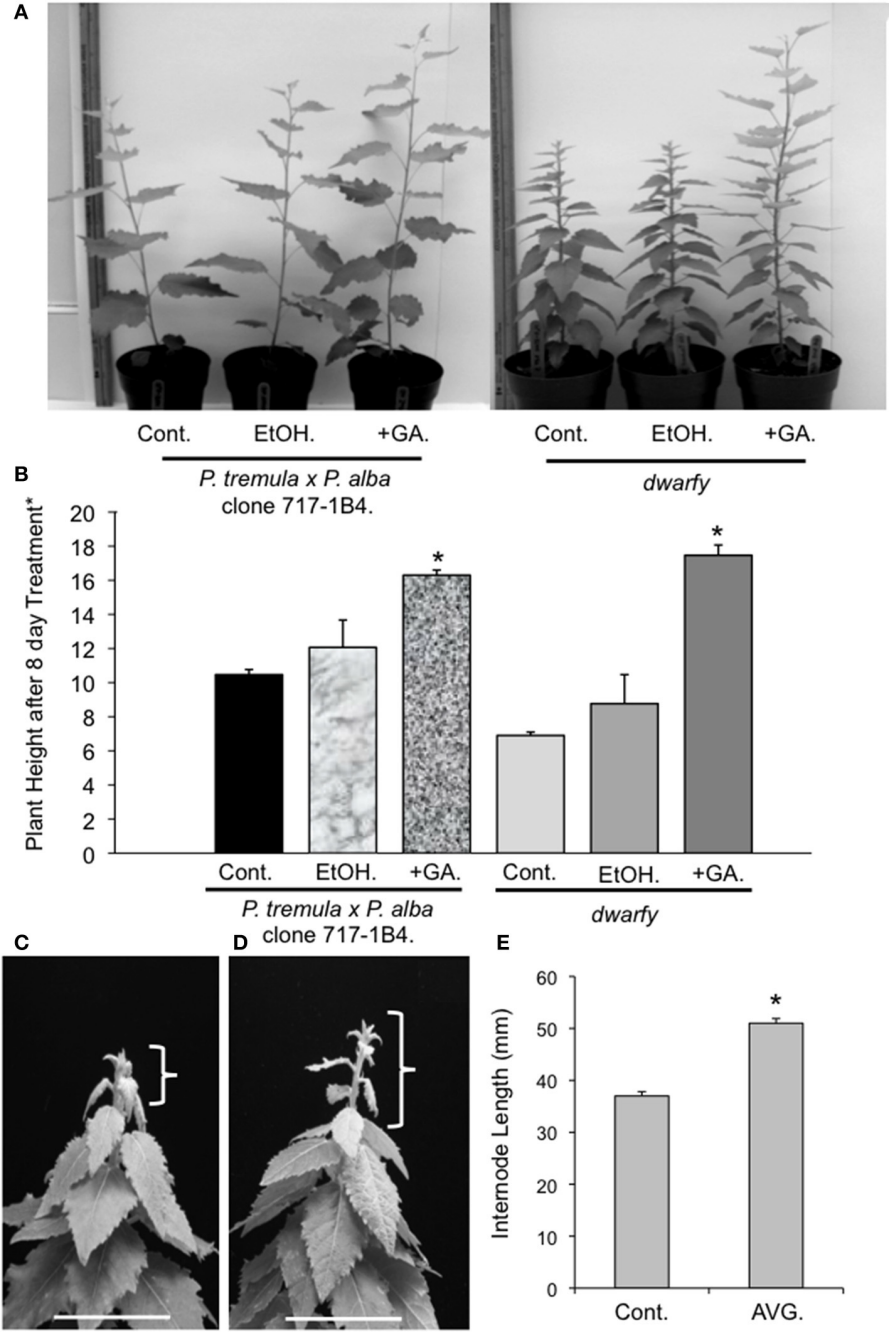
**Application of GA and AVG to *dwarfy* apexes induces faster growth rate. (A)** Representative image of GA influence on the growth rates of *dwarfy* and wildtype (+GA) as compared to ethanol control (+EtOH) and untreated control (Cont.). **(B)** Mean heights of wild-type and *dwarfy* saplings treated with GA (+GA) as compared to ethanol control (+EtOH) and untreated control (Cont.). **(C)** Representative image of water and **(D)** AVG influence on the growth rates of *dwarfy* and wild-type. Parentheses indicate growth of main stem for the treatment period. Scale bar = 3 cm. **(E)** Mean internode lengths wild-type and *dwarfy* saplings treated with AVG as compared to water control (Cont.). All values ±SE. ^*^Significantly different from wildtype (*p* < 0.05).

**Figure 5:**
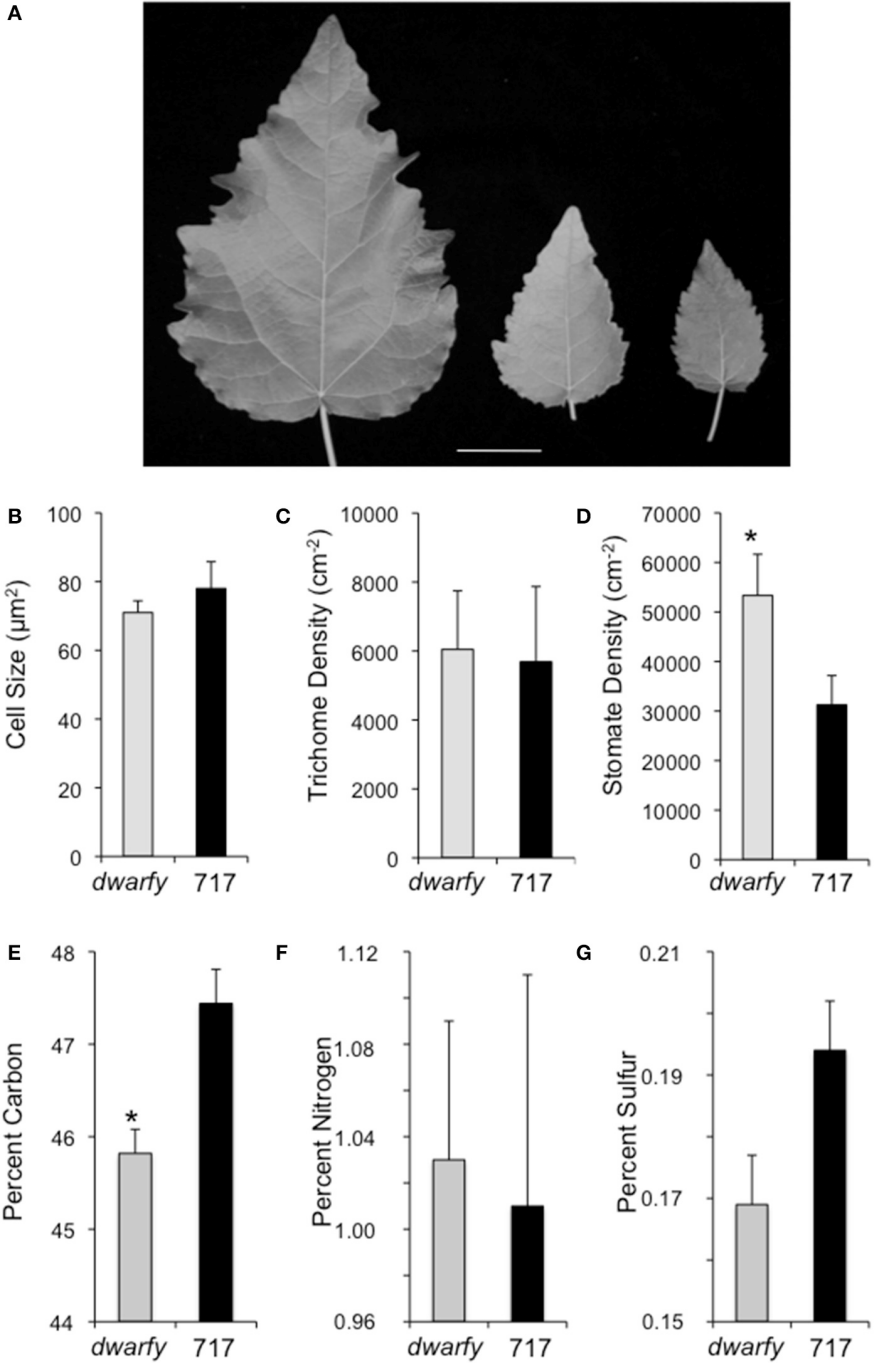
**Elevated expression levels of *PtaACS8* result in significant changes in leaf architecture and physical characteristics. (A)** A representative image of fully expanded leaves of wildtype, one independent line of the *35S::PtaACS8* mutant *P. tremula x P. alba* clone 717-1B4 and *dwarfy*, respectively, after 2 months of growth. Scale bar = 2 cm. Epidermal cell density **(B)**, trichome density **(C)** and stomate density **(D)** in fully expanded leaves of *dwarfy* (gray bars) as compared to wild-type leaves (black bars). Relative percentage of carbon **(E)**, nitrogen **(F)**, and sulfur **(G)** in mature leaves of wildtype (black bars) and *dwarfy* (gray bars). All values ±SE. ^*^Significantly different from wildtype (*p* < 0.05).

**Figure 6:**
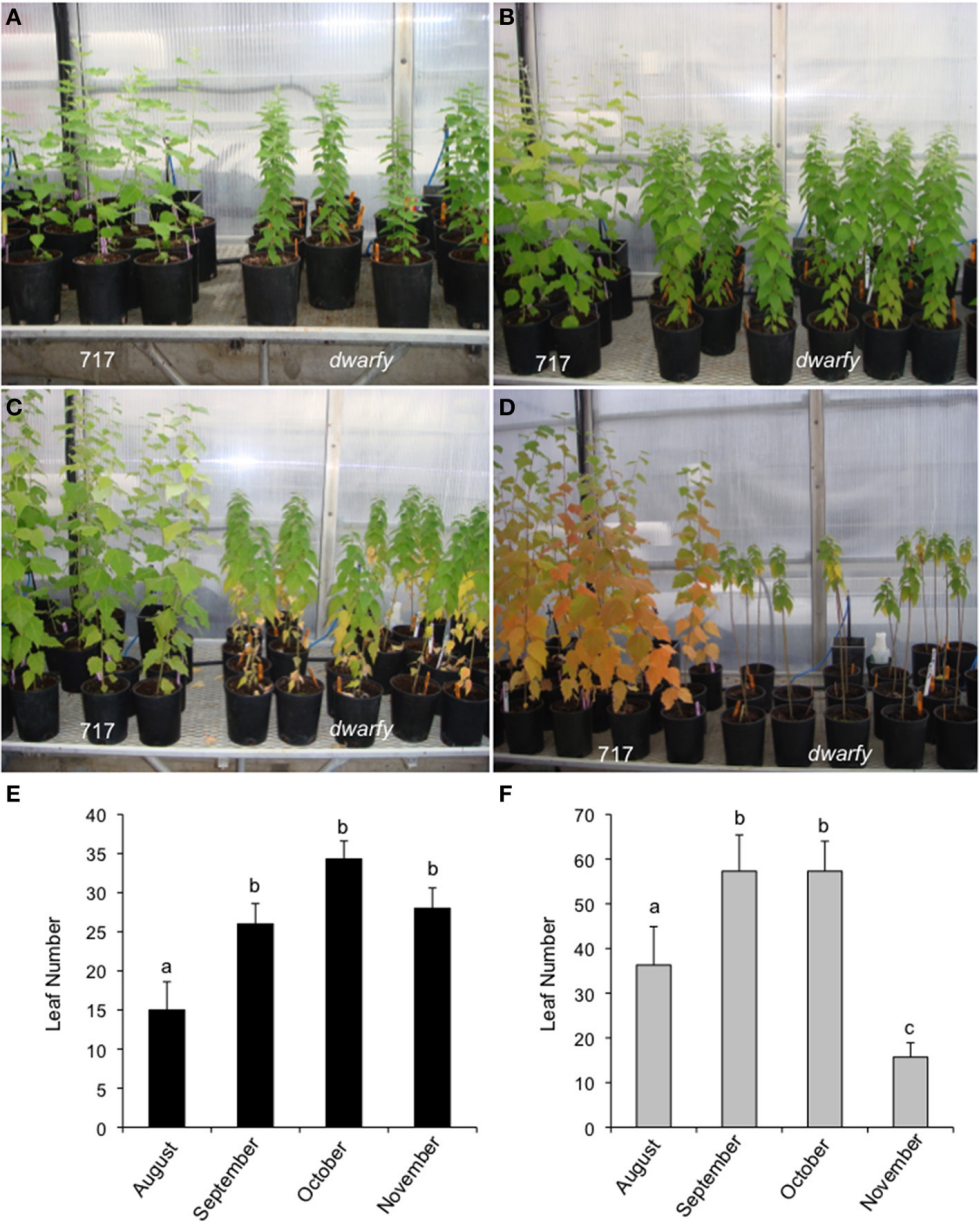
***dwarfy* mutants exhibit pre-mature leaf senescence**. Comparison of leaf senescence rates in wildtype and *dwarfy* mutant clones within their first year of growth in August **(A)**, September **(B)**, October **(C)**, and November **(D)**. Leaf numbers on wild-type trees **(E)** and *dwarfy* trees **(F)** over the same time period are presented. ±SE; superscript letters indicate significant differences between treatments as determined by One-Way analysis of variance (ANOVA) followed by a Tukey HSD (honestly significant difference) multiple comparison test (*p* < 0.05).

All plants used in Figures 1, **4**, **6** were grown under greenhouse conditions at the Canadian Forest Service (CFS), Fredericton, New Brunswick, Canada, while plants used for data analysis in Figures 2, 3, **5** were grown under greenhouse conditions at Queen's University, Kingston, Ontario, Canada. In the former situation, plants were grown under natural daylight and temperature, while in the latter situation, photoperiod was maintained at 16 h per day and temperature at 25°C. The *dwarfy* mutant was generated as described by Harrison et al. (2007) in a *P. tremula x P. alba* clone 717-1B4 background and all comparisons of *dwarfy* were made with this hybrid (wildtype). The *dwarfy* mutant was initially identified based on the dwarf characteristics such as plant height and leaf size among others in the mutant.

### T-DNA insertion analysis

Southern analysis of the *dwarfy* poplar mutant line was done and confirmed the presence of one T-DNA insertion event (Harrison et al., 2007). Genomic DNA (gDNA) was extracted from CFS greenhouse *dwarfy* mutant leaves using the cetyltrimethylammonium bromide (CTAB) method and gDNA was quantified using an Nanodrop1000 spectrophotometer and quality was checked on 0.8% (w/v) agarose Tris-acetate EDTA ethidium bromide gel. To identify the site of T-DNA insertion, the Genome Walker™ universal kit (Clontech, http://www.clontech.com) was used according to the manufacturer's protocol to create 4 restriction digested gDNA libraries. Each library was analyzed by primary and nested PCR using T-DNA vector specific primers (VSP 1 and VSP 2) designed from the T-DNA sequence and adapter primers AP1 & AP2 provided in the kit (Table S1). Primary PCRs were done on 1 μl of each library except using High Fidelity Platinum Taq (Invitrogen) for the PCR reaction mix. Primary PCR reactions from each library were diluted 50 times in H_2_O and 1 μl of the each dilution was used for nested PCR analysis using the same PCR reaction components except primers VSP2 & AP2 were used. Cycling parameters for both primary & nested PCRs were the same ones stated in the kit except that the elongation time was increased to 5 min. Primary and nested PCRs were analyzed by gel electrophoresis and bands from the nested PCR reaction that were over 1 kb in size were subcloned in pCR4-topo vector using the TOPO TA cloning kit (Invitrogen, http://www.invitrogen.com) and fully sequenced at the McGill University and Genome Quebec Innovation Centre (http://gqinnovationcenter.com). Based on flanking sequence information, a flanking gDNA primer FSP1 was designed and PCR was done on *dwarfy* gDNA using FSP1 and VSP 2 primers to confirm the location of the T-DNA insertion site. This amplicon was TOPO TA cloned and fully sequenced. Localisation of insertion site was determined by BLASTn using flanking sequence as query against the *P. trichocarpa* genome (Tuskan et al., 2006) (*Populus trichocarpa* v3.0, DOE-JGI, http://www.phytozome.net/poplar).

### Affymetrix array analysis

For gene expression analysis, total RNA was extracted from 0.5 g of different tissues of wildtype and *dwarfy* taken from CFS greenhouse grown plants using a modification of Chang et al., 1993 and the RNeasy kit (QIAGEN). Total RNA quality and quantity was determined by Nanodrop1000 and by electrophoresis. Triplicate samples of *dwarfy* and wild-type leaf and stem total RNA were sent to the Microarray Centre (University Health Network, Toronto (UHN)) for sample processing and analysis. Sample quality was verified with the Agilent 2100 Bioanalyser before analysis with the GeneChip® Poplar Genome Array. Data was also analyzed by the Microarray Center (UHN) using Gene Spring software.

### Isolation of ACS8 candidate gene coding sequence

The *ACS8* cDNA was isolated from wild-type leaf tissues using the Smart RACE kit (Clontech, http://www.clontech.com) according to manufacturer's protocol. One microgram of total RNA was used to produce the 5′&3′RACE ready products and *ACS8* gene specific primers ACS8RACE.fwd and ACS8RACE.rev were used along with the Universal Primer (UP) provided in the kit (Table S1). 5′&3′ RACE products were subcloned in pCR4-topo vector using the TOPO TA cloning kit and sequenced. Gene specific primers; ACS8-ATG.fwd and ACS8-Stop.rev primers were designed and used to isolate the full *ACS8* CDS using the 3′RACE ready product previously generated and the amplicon was cloned in pBluescript II (+) (Fermentas, http://www.fermentas.com) using *Hin*dIII-*Xba*I restriction sites. The resulting construct carrying the full *ACS8* CDS was fully sequenced.

### Agrobacterium tumefaciens mediated tranformation of *P. tremula x P. Alba* clone 717-1B4

In order to generate a binary plant transformation vector, *ACS8/*pBluescript II (+) construct was digested with *Eco*RI and subcloned in pART7 (Gleave, 1992) and verified by restriction digest for correct orientation between the CaMV promoter and ocs 3′ region. The *ACS8*/pART7 construct was subsequently digested with NotI and the whole cassette was ligated in the binary vector pART 27 (Gleave, 1992) prior to *Agrobacterium tumefaciens* transformation in line 717-1-B4 (Harrison et al., 2007). Out of 17 independent transgenic lines generated, 6 lines survived the transfer to greenhouse conditions and these lines were analyzed. Total RNA from leaf tissue was extracted from newly transformed lines and RT-qPCRs were done for gene expression analysis of lines generated using the procedures as stated below. Each line was analyzed in duplicate technical replicates. Internode lengths were measured after 3 months of growth.

### Quantitative gene expression analysis

For in gene expression analysis in transgenic 35S::*PtaACS8* lines, total RNA was extracted from 100 mg of shoot apical tissue using the RNeasy kit (QIAGEN). Total RNA quality and quantity was determined by Nanodrop1000 and by electrophoresis. Two to four micrograms of total RNA was treated with Turbo DNaseI (Ambion, http://www.ambion.com) and RT-qPCR was done with 50 ng of total RNA/reaction using the one step Quantitect SYBR Green RT-PCR kit (QIAGEN, http://www.qiagen.com). RT-qPCR cycling conditions were: 30 min at 50°C for reverse transcriptase reaction and 15 min at 95°C for enzyme inactivation followed by 40 cycles of 15 s at 94°C, 15 s denaturation at 55°C (annealing) and 30 s at 72°C elongation followed by fluorescence measurement. The relative expression of *PtaACS8* was compared to the *UBQ10* reference gene (Plett et al., 2010).

The amplification efficiencies of each gene primer set were determined by *E* = 10^[−1/slope^] and were calculated using the slopes of n-fold serial dilution standard curves. Fold change ratios were determined using the comparative Ct method (ΔΔCt method) since amplification efficiencies were approximately equal in all target and reference genes measured in the study. Samples were analyzed in triplicates of each wildtype and the *dwarfy* mutant plants. Each total RNA sample was analyzed in duplicate. A No Reverse Transcriptase (NoRT) for each sample was included and a No Template Control (NTC) was included for each primer pair to make sure no contamination was present in the experiments. Amplicon specificity was confirmed by electrophoresis (single band at the right size), by melt curve analysis (single peak and Tm) and by sequencing.

### Ethylene determination

Leaf and stem samples were removed from wild-type and *dwarfy* poplar plants between 10 AM and 12 PM, and incubated in 20 mL headspace vials for 4 h at ambient temperature. Fresh weight was recorded and time between vial seal and sample injection were noted to have an exact incubation time. Ethylene content within this headspace was determined by gas chromatography coupled to a flame ionization detector (Gas Chromatography- Flame Ionization Detector GC-FID, Agilent 7890A) with an injector (splitless mode) temperature of 240°C and oven temperature at 60°C (isothermal) using helium as carrier gas (3 mL/min). A30 m × 0.53 mm ID (30 μm average thickness) Carboxen 1006 PLOT column (Supelco, Sigma Aldrich, Ontario Canada) was used to separate ethylene from the mixture. The FID (heated to 240°C) hydrogen:air:makeup flows were 30:400:25 (mL/min). Two measurements of 0.1 mL gas aliquot was taken from a headspace vial using a gas tight syringe (Hamilton 1700 series) and immediately injected for each sample. At these conditions, the observed retention time of ethylene was 3.14 min. A calibration curve was generated to cover the range of 0.1–20 mg ethylene. Ethylene standard gas mixture was made by drawing a volume of 99.5% ethylene (Praxair) and injecting it in a previously vacuum-purged sealed headspace vial (volume determined by water capacity), then breaking the vacuum with a syringe needle and filling the vial with ambient air to atmospheric pressure. The diluted ethylene was allowed to stand for 1 h to reach dispersal equilibrium. Increasing volumes were injected to cover the desired ethylene range and each injection was repeated in triplicate.

### Physical characteristics analysis

Cuttings of wildtype and *dwarfy* were established by cutting 4–5 cm shoot explants from stock plants. Cuttings were planted in Jiffy 42 mm peat plugs grown under greenhouse conditions June to August (natural lighting, watered twice daily) for 7 weeks. After 7 weeks, cuttings were transferred to greenhouse pots (15 cm diameter, 19 cm long). After a total of 9 weeks, 3 trees were randomly selected every month and height was measured and data analyzed using basic statistical tools in Excel (Microsoft Office). Leaf cell size, trichome density and cell density were performed as per Plett et al. (2010). Fiber and vessel isolation and measurements were performed as per Chaffey et al. (2002).

### GA and ethylene biosynthetic inhibitor growth effect analysis

Plants used for GA effect on growth were grown under normal greenhouse conditions as mentioned above. A triplicate (for GA) or a duplicate (for AVG) of wild-type and *dwarfy* plants of similar heights, grown for 5 weeks from cuttings, were used for each treatment for the experiment. Plant height was measured before the experiment and measured prior to each new application of GA or AVG. A total of 3 applications of 10 μl of 3 mM GA/water or ETOH (for GA analysis) or of water or 100 μM AVG were added every fourth day to the shoot apex of each plant and the total length of the experiment was 12 days. Data was analyzed using the height difference between the first measurement (before first application) and before 3rd application (3rd measurement), since 2 of the dwarfy/GA treated shoot apex samples had dried up and were dead before the final measurement.

### Percent carbon, nitrogen and sulfur analysis

Dried leaf, stem, and roots samples from both wildtype and *dwarfy* were ground with a bead mill, and kept under vacuum in order to keep moisture out of the samples prior to carbon (C), nitrogen (N) and sulfur (S) analysis (CNS) by the CFS analytical laboratory according to the method of Kalra and Maynard (1991). A triplicate of each clone for each tissue types was measured and data was analyzed using basic statistical tools in Excel (Microsoft Office). Results presented are the measure of C, N and S from healthy mature leaves and internode tissues harvested in June of the growing season.

## Results

### Endogenous over-expression of PtaACS8 induces dwarfism in populus

From a large population of activation-tagged *P. tremula x P. alba* clone 717-1B4 (2000 independent transgenic lines; Harrison et al., 2007), we identified one line with a consistent reduction in growth rate over multiple growing seasons (Figures 1A,B). This mutant was named *dwarfy*. Using Southern blotting only one T-DNA insert in *dwarfy* and located this insert on chromosome 2 using a modified TAIL PCR was identified. Within a window of ±20 Kb around the T-DNA, 3 genes annotated in Phytozome (Figure 1C) were found as follows: a gene of unknown function (Potri.002G11400; +14.4 Kb up-stream), *PtaACC SYNTHASE8* (*PtaACS8;* Potri.002G113900; 13.1 Kb down-stream) and *PtaEARLY-RESPONSE TO DEHYDRATION 4* (*ERD4;* Potri.002G113800; 16.9 Kb down-stream). A quantification of the expression of these genes in the *dwarfy* mutant line relative to wild-type *P. tremula x P. alba* clone 717-1B4 demonstrated that only *PtaACS8* exhibited increased gene expression in all aerial tissues of the plant (Figure 1D). As ACC synthases are involved in the first step in the biosynthesis of the plant hormone ethylene, ethylene production was measured in the same three compartments as used for gene expression analysis in wild-type and mutant plants (i.e., young and mature leaves and stem tissues). Compared to wildtype, the mutant line produced 14× higher levels of ethylene in younger leaves and 6× higher levels of ethylene in mature leaves and the stems (Figure 1E).

To verify that increased transcript abundance of *PtaACS8* was indeed responsible for the dwarfism phenotype of the mutant, the Potri.002G113900 gene was cloned and expressed ectopically in the *P. tremula x P. alba* clone 717-1B4 genetic background under the control of the 35S-cauliflower mosaic virus promoter. We were able to regenerate six independent transgenic lines from callus culture which, when grown alongside age-equivalent wildtype (i.e., propagated at the same time and treated in the same manner as the *35S::PtaACS8* lines), displayed a dwarf phenotype (Figure 2A). This reduction in growth and internode length was significant as compared to wildtype in all lines tested although the plants were consistently bigger than *dwarfy* (Figures 2A,B). The discrepancy in height difference is likely due to the fact that none of the *35S::PtaACS8* transgenic lines displayed the same level of *PtaACS8* transcript accumulation as *dwarfy* (Figure 2C). As there was a significant correlation between the transcript abundance of *PtaACS8* and the dwarf phenotype in the transgenic lines (Figure 2D; *r* = 0.91; *p* < 0.001), we conclude that increased transcript abundance of *PtaACS8* in the original *dwarfy* transgenic line is responsible for the reduction in plant stature.

### Increased transcript abundance of PtaACS8 leads to altered stem characteristics

The *dwarfy* mutant line exhibited alterations to the morphology of all aerial parts of the plant. While the internode length of the *dwarfy* line was significantly reduced (Figure 2B), there were also significant alterations to the microscopic anatomy of the stem (Figure 3). Due to the great difference in height of the two plants being compared, we used a plastochron index to identify and compare the same internode between the mutant line and wildtype. We used different microscopy techniques to observe different stem properties: brightfield to gain a general over-view of the stem architecture, UV excitation to differentiate chlorophyll autofluorescence (red signal) from secondary cell wall fluorescence (blue-green signal; Figures 3A,C). In young stems (internode between leaves 10 and 11), there was a reduction in the amount of secondary xylem formed in *dwarfy* as compared to wildtype (Figure 3A) as well as a significant reduction in the length of xylem fibers and vessels (Figure 3B; *p* < 0.05). In older stem tissues (internode between leaves 20 and 21), the reduction in secondary xylem formation (Figure 3C) and fiber/vessel lengths were still observed (Figure 3D). Detailed analysis of wood formation in these older tissues also revealed a difference in cell density: *dwarfy* had a higher density of xylem vessels per square millimeter with a significantly smaller outer diameter as compared to wild-type stems (Figures 3A,C,E–G) while there was no significant difference in the density of fibers. As alterations to the cell make-up of the stem and alteration in growth rate may influence nutrient deposition in the stem, we analyzed the percentage of carbon, nitrogen and sulfur in these mature internodes of both wildtype and *dwarfy*. No significant difference in percent accumulation of carbon and sulfur in the stems of *dwarfy* and wildtype were observed while the stems of the former accumulated a significantly higher concentration of nitrogen-containing compounds (Figures 3H–J; *p* < 0.05).

### Hormone- and nutrient-related genes display altered abundance in *dwarfy* stems

In order to understand the transcriptomic profile of *dwarfy* stems, we performed a whole genome oligo-array transcriptomic analysis of whole stem tissues. We found 223 genes differentially expressed (≥2-fold; *p* < 0.05) as compared to wild-type *P. tremula x P. alba* clone 717-1B4 stems of the same age (Table S2). Within these genes we found that there were a number of ethylene and auxin related genes and genes coding for proteins involved in nutrient transport and biosynthesis (Table 1). Genes related to the ethylene pathway included *PtaACS8* (>230-fold increase) a number of ETHYLENE RESPONSE FACTOR (ERF) genes, two serine-threonine receptor kinases (*PtaCTR3, PtaCTR4*) and two ethylene receptor genes (*PtaETR1, PtaETR5*). The majority of genes associated with the auxin pathway, meanwhile, were repressed in the stems of *dwarfy* while a gene encoding an IAA-amido-synthetase glycosyl-hydrolase (GH) family protein displayed increased abundance. Nutrient transport and synthesis was also affected with two sugar transporters and an amino acid transporter being repressed while the transcript accumulation of a glutamine synthase was increased (Table 1).

**Table 1:** **Genes found to have significantly different abundance in the stems of *dwarfy* as compared to wildtype**.

**Probe**	**RefSeq protein ID**	***E*-value**	**Fold change**	**Gene title**
**ETHYLENE RELATED**				
PtpAffx.202003.1.S1_at	XP_002302380	0.00E+00	233.9	1-Aminocyclopropane-1-carboxylate 8 (*ACS8*)
Ptp.6619.1.S1_s_at	XP_002315490	8.00E–144	30.5	AP2/ERF domain-containing transcription factor
PtpAffx.75787.1.A1_s_at	XP_002297877	0.00E+00	20.4	AP2/ERF domain-containing transcription factor
PtpAffx.75787.1.A1_at	XP_002304640	0.00E+00	14.3	AP2/ERF domain-containing transcription factor
PtpAffx.129036.1.S1_at	XP_002316302	1.00E–16	12.3	Ethylene-responsive protein
PtpAffx.219707.1.S1_at	XP_002326299	0.00E+00	8.1	AP2/ERF domain-containing transcription factor
PtpAffx.4624.1.S1_at	XP_002328620	0.00E+00	5.8	AP2/ERF domain-containing transcription factor
PtpAffx.572.3.S1_a_at	XP_002315958	0.00E+00	5.3	AP2/ERF domain-containing transcription factor
Ptp.162.1.A1_at	XP_002302732	0.00E+00	3.6	Ethylene receptor 1 (*PtETR1*)
PtpAffx.79014.1.S1_at	XP_002316514	0.00E+00	3.5	Serine/threonine protein kinase (*PtCTR4*)
Ptp.866.1.S1_s_at	XP_002310408	4.00E–118	3.3	AP2/ERF domain-containing transcription factor
PtpAffx.208193.1.S1_at	XP_002311669	0.00E+00	2.8	Ethylene receptor 5 (*PtETR5*)
PtpAffx.122897.1.A1_at	XP_002308565	1.00E–96	2.7	*REVERSION-TO-ETHYLENE SENSITIVITY1* (*RTE1*)
PtpAffx.13062.4.S1_at	XP_002308982	0.00E+00	2.5	ein3-binding f-box protein 4
Ptp.2044.2.S1_a_at	XP_002311967	0.00E+00	2.3	Serine/threonine protein kinase (*PtCTR3*)
**AUXIN RELATED**				
PtpAffx.144034.1.S1_s_at	XP_002310372	1.00E–06	3.2	*AUXIN-REGULATED GENE INVOLVED IN ORGAN SIZE* (*ARGOS*)
Ptp.6069.1.S1_at	XP_002320183	0.00E+00	2.9	GH3 family protein
Ptp.8069.1.S1_at	XP_002306504	0.00E+00	–2.1	*NAKED PINS IN YUC MUTANTS 2* (*NPY2*)
PtpAffx.155898.1.S1_at	XP_002320550	5.00E–109	–2.3	Dopamine beta-monooxygenase
PtpAffx.97214.1.A1_at	XP_002302687	2.00E–132	–2.3	Auxin-induced protein 5NG4
PtpAffx.117529.1.S1_at	XP_002323866	0.00E+00	–3.0	MDR family ABC transporter family
PtpAffx.210100.1.S1_at	XP_002317029	2.00E–157	–3.1	Auxin:hydrogen symporter
PtpAffx.7696.4.S1_at	XP_002312567	1.00E–75	–4.3	Auxin-responsive protein IAA4
**NUTRIENT SYNTHESIS/TRANSPORT**				
PtpAffx.2311.1.S1_s_at	XP_002313246	0.00E+00	6.2	*GLUTAMINE-DEPENDENT ASPARAGINE SYNTHASE 1* (*ASN1*)
PtpAffx.217242.1.S1_at	XP_002331420	3.00E–169	–2.3	Sugar transporter
Ptp.5882.1.S1_at	XP_002301819	3.00E–43	–2.1	RS21-C6 protein
PtpAffx.111624.1.S1_at	XP_002302894	0.00E+00	–10.2	Amino acid transporter

*(p < 0.05; >2-fold differential regulation). Note: In this table there are no column lines as there are in Table 2*.

GA has been linked to enhanced growth phenotypes through the induction of auxin biosynthesis and polar transportation (Björklund et al., 2007). Therefore, as our transcriptional analysis of the *dwarfy* mutant indicated that auxin transport and signaling was affected, we tested whether GA application to the growing apex of *dwarfy* would be able to rescue the growth phenotype of the mutant. We found that the growth rate of *dwarfy* was significantly increased by treatment with GA (Figures 4A,B). Therefore GA is able to rescue the *dwarfy* phenotype. We also treated *dwarfy* with the ethylene biosynthetic inhibitor AVG. This treatment resulted in an increase in internode length (Figures 4C–E), demonstrating that blocking ethylene synthesis also rescues the *dwarfy* phenotype.

### Increased transcript abundance of PtaACS8 leads to altered leaf characteristics

Mature leaves in the *dwarfy* mutant also showed altered size when compared to wild-type leaves (Figure 5A). The leaves of *dwarfy* were much smaller than those of wildtype (Figure 1A). Despite the alterations in leaf size, the epidermal cell size of *dwarfy* was not significantly altered compared to wildtype (Figure 5B). Trichome density was also not affected, but stomate density was significantly higher in the *dwarfy* mutant (Figures 5C,D). Only nitrogen content was significantly higher in *dwarfy* stems, as compared to wildtype (Figure 3I). Unlike stems, the total percentage of carbon in leaves was significantly reduced in *dwarfy* as compared to wildtype (Figure 5E), while nitrogen levels were not altered (Figure 5F). Sulfur levels in mutant leaves showed a tendency toward a lower accumulation compared to wildtype, but this difference was not found to be significant (Figure 5G; *p* < 0.05). The date at which leaves became chlorotic and dropped off the stem in *dwarfy* as compared to wild-type plants was assessed as increased ethylene levels have been correlated to early leaf senescence (Breeze et al., 2011; Koyama et al., 2013). When grown under natural conditions, chlorosis of 1-year-old *dwarfy* leaves happens earlier as compared to wild-type plants (Figures 6A–D) and significant leaf drop occurred in *dwarfy* plants in the month of November while there was no significant leaf drop in the same period in wild-type trees (Figures 6E,F). It is interesting to speculate that the reduced C and S observed in the *dwarfy* leaves may be related to the shorter growing season for these leaves.

### Senescence- and cell cycle-related genes exhibit altered abundance in *dwarfy* leaves

We found that 183 genes were significantly regulated in fully expanded leaves of *dwarfy* as compared to wild-type *P. tremula x P. alba* clone 717-1B4. A large number of hormone-related genes with altered transcription were observed in the stems of *dwarfy*, while only two of these genes (*PtaACS8* and a *GH3* family protein) were significantly differentially regulated in mature *dwarfy* leaves (≥2-fold; *p* < 0.05; Table 2; Table S3). A number of nutrient transporters displayed altered transcript abundance, although they were different from those identified in *dwarfy* stems (Table 1). Three other classes of genes were differentially regulated in mature *dwarfy* leaves that were not observed in the stems: defense-, senescence- and cell cycle/expansion-related genes (Table 2). The majority of the defense-related genes were associated with pathogen attack, including a chitinase, a lipase and a glyoxal oxidase. As the leaf tissues were healthy at the time of harvest and displayed no infection structures, the activation of these genes is likely constitutive in the *dwarfy* background. Three genes associated with leaf senescence were also up-regulated. One group that only showed reduced levels of transcript abundance was that of genes associated with cell cycle and cellular growth (Table 2). Within this group of genes were a number of cyclins, calmodulin-like proteins and one expansin.

**Table 2:** **Genes found to have significantly different abundance in the leaves of *dwarfy* as compared to wildtype**.

**Probe**	**RefSeq protein ID**	***E*-value**	**Fold change**	**Gene title**
**HORMONE**				
PtpAffx.202003.1.S1_at	XP_002302380	0.00E+00	506.5	1-Aminocyclopropane-1-carboxylate 8 (*ACS8*)
PtpAffx.211163.1.S1_s_at	XP_002319260	0.00E+00	2.8	GH3 family protein
**NUTRIENT TRANSPORT**				
PtpAffx.79594.1.S1_s_at	XP_002318842	8.00E–164	3.2	Sorbitol dehydrogenase-like protein
PtpAffx.15690.1.S1_at	XP_002311043	0.00E+00	3.0	Proline transporter
Ptp.3435.2.S1_s_at	XP_002302223	0.00E+00	2.6	Amino acid permease
Ptp.1552.1.S1_s_at	XP_002302727	0.00E+00	2.1	SUS3 (sucrose synthase 3)
Ptp.8110.1.S1_at	XP_002313213	0.00E+00	–3.5	Oligopeptide transporter
**DEFENSE**				
PtpAffx.77318.1.S1_x_at	XP_002312918	1.00E–114	10.4	Chitinase
PtpAffx.50871.1.A1_at	XP_002302379	0.00E+00	5.8	Lipase
Ptp.6139.1.S1_at	XP_002304920	0.00E+00	4.8	Cytochrome P450
PtpAffx.136901.1.S1_at	XP_002306296	3.00E–165	3.6	GCL1-like
Ptp.2230.1.S1_at	XP_002302409	5.00E–133	3.2	Sigma factor B
PtpAffx.55005.1.A1_at	XP_002322929	0.00E+00	2.8	Glyoxal oxidase-related
**SENESCENCE**				
PtpAffx.57533.1.S1_a_at	XP_002320492	6.00E–165	37.0	Triacylglycerol lipase 2 precursor
Ptp.2629.1.S1_s_at	XP_002307593	2.00E–40	2.7	Senescence-associated protein-related
PtpAffx.85571.1.S1_s_at	XP_002299638	1.00E–31	2.2	Senescence-associated protein-related
**CELL CYCLE/EXPANSION**				
PtpAffx.222953.1.S1_at	XP_002318886	0.00E+00	–2.3	Trehalose-6-phosphate synthase
PtpAffx.50897.2.S1_at	XP_002310432	3.00E–55	–2.3	Calmodulin 24-like protein
PtpAffx.200879.1.S1_at	XP_002298451	2.00E–156	–2.4	Cyclin
Ptp.5638.1.S1_at	XP_002307791	0.00E+00	–2.5	Cyclin B
Ptp.7389.1.S1_at	XP_002319120	0.00E+00	–2.5	CDC20.1
PtpAffx.162051.1.S1_a_at	XP_002322260	3.00E–35	–2.8	CDC2-like protein kinases
Ptp.1602.1.S1_at	XP_002307822	1.00E–177	–2.9	Cyclin-dependent kinase
PtpAffx.63679.1.A1_s_at	XP_002306649	0.00E+00	–4.0	Cyclin A
Ptp.2869.1.A1_at	XP_002299019	0.00E+00	–4.2	Patellin-4
PtpAffx.206669.1.S1_s_at	XP_002308551	0.00E+00	–4.6	Calmodulin binding protein
PtpAffx.17914.3.A1_at	XP_002312101	7.00E–140	–4.8	Expansin

*p < 0.05; >2-fold differential regulation*.

## Discussion

Due to ornamental value and to wide-ranging applications within agriculture, the genetic traits that control cell size and dwarfism in plants have been widely studied (Valdovinos et al., 1967; Ephritikhine et al., 1999; Busov et al., 2003; Qi et al., 2011; Luo et al., 2013; Li et al., 2014; Yang et al., 2014; Zhang et al., 2014). Through breeding practices and applications of growth regulators, dozens of different dwarf plant varieties have been produced over the past few decades (Parker, 2012; Jiang et al., 2013; Wang et al., 2014a,b). Largely studied in annual models such as *Arabidopsis, Zea* and *Oryza*, control of plant stature has been linked most readily to plant hormones. For example, *Arabidopsis* mutants with increased ethylene production (e.g., *eto;* Woeste et al., 1999) exhibit thickening of the hypocotyl while increased ethylene signaling (e.g., *ctr1-1; ACS6^DDD^*; Liu and Zhang, 2004) has been found to result in reduced stature and smaller leaf size. Due to advancements in insertional mutagenesis and other transgenic technologies, it is now becoming feasible to also screen perennial plants for the genes that control dwarfism (Busov et al., 2003; Harrison et al., 2007; Vahala et al., 2013). Here we characterize a mutant line of *P. tremula x P. alba* clone 717-1B4 that exhibits higher transcript accumulation of *PtaACS8* and that produces a significantly higher level of ethylene in all aerial tissues as compared to wild-type trees. Increased transcript abundance of *PtaACS8* is correlated to reduced plant stature and smaller leaves while treatment of *dwarfy* shoots induces increases in internode length. The transcriptional cascade induced by altered levels of *PtaACS8* are very different in stem and leaf tissue with a transcriptional reduction in genes associated with auxin transport and signaling evident in stems and repressed cell cycle genes in the leaves. These results place *PtaACS8*, and likely ethylene, as regulators that control two major morphological traits associated with dwarfism and reduced tissue size.

Using transformation technologies such as activation tagging is a very useful approach to identifying and characterizing the role of genes in a physiologically relevant manner. Rather than ectopic over-expression of a gene, the inserted enhancer used in activation tagging only enhances expression in its native expression pattern. This mutagenesis approach has been used in a number of model plant systems including *Arabidopsis* (Weigel et al., 2000), tomato (Mathews et al., 2003), rice (Jeong et al., 2006), and poplar (Harrison et al., 2007). Using this approach Busov et al. (2003) were able to identify a poplar *GA2-OXIDASE* that resulted in a plant with a very similar phenotype to that described here for *dwarfy;* reduced stature and smaller leaves. Since their publication, dwarfism in a native dwarf plum tree cultivar has also been linked to a *GA2-OXIDASE* (El-Sharkawy et al., 2012) demonstrating that findings from activation tagging studies can be extended to natural plant populations.

As opposed to a strictly GA-dependent phenotype, our results support the hypothesis that growth retardation in *dwarfy* is driven largely by ethylene, the endpoint of the biochemical pathway in which *PtaACS8* operates. This is based on the evidence that increased *PtaACS8* transcripts are correlated to significant increases in ethylene production in the stem (Figure 1E), whose signal is being relayed by the activation of several ERF genes (Table 1). Our results also demonstrate that blocking of ethylene biosynthesis resuces the *dwarfy* phenotype (Figures 4C–E). Further, the reduction in xylem fiber and vessel length described here-in has also previously been observed after ethylene treatment of poplar stems (Junghans et al., 2004; Love et al., 2009; Vahala et al., 2013). While we cannot rule out the possibility that the reduced stature in *dwarfy* is a result of increased ACC accumulation, our results support the hypothesis that stunting of the *dwarfy* stem is controlled in an ethylene-dependent manner. Increased ethylene, however, is likely not the only causative factor in explaining the stature of *dwarfy*. Rather, ethylene appears to be influencing another pathway associated with plant stature: the auxin pathway. We found evidence for a repression of auxin-homeostasis and transport genes in the stem of *dwarfy* (Table 1). Ethylene has long been tied to a negative effect on auxin diffusion (von Guttenberg and Steinmetz, 1947; Morgan and Gausman, 1966; Valdovinos et al., 1967; Suttle, 1988; Andersson-Gunneras et al., 2003; Ruzicka et al., 2007; Stepanova et al., 2007; Swarup et al., 2007). As inhibition of auxin diffusion has been correlated to a reduction in stem cell elongation of poplar (Junghans et al., 2004), pea (Lantican and Muir, 1969), tomato (Higashide et al., 2014), tulip (Okubo and Uemoto, 1985), *Arabidopsis* (Franklin et al., 2011; Chae et al., 2012), gourds (Wang et al., 2014a) amongst many other systems. Our results give a molecular framework by which ethylene affects *dwarfy* height where increased expression of *PtaACS8* results in greater production of ethylene which, upon perception in plant stem tissue, represses genes related to auxin diffusion and synthesis which would then curtail cell elongation in the stem. GA treatment of growth apexes can also rescue the *dwarfy* phenotype, although we cannot conclude from present data if GA generates this phenotype by acting downstream of the ethylene signal in the *dwarfy* mutant or in a separate pathway.

A different genetic pathway is likely responsible for the observed reduction in leaf size in *dwarfy*. While increases in *PtaACS8* transcripts and ethylene evolution in the stem coincided with stunted fiber and vessel growth, no change in leaf epidermal cell size is observed despite higher levels of *PtaACS8* transcripts and higher ethylene evolution in the leaves. This would indicate that the leaf is smaller due to the absolute number of cells making up the tissue rather than the size of cell generated. It is interesting in the leaves of *dwarfy* that we see no evidence of compensation by leaf epidermal cells to maintain a larger leaf area. “Compensation” occurs when upstream inhibition of cell division initiates a secondary signaling pathway that increases cell size to maintain proper tissue growth (Hemerly et al., 1995; DeVeylder et al., 2001; Tsukaya, 2002; Horiguchi et al., 2006). Ethylene treatment has been associated with both stimulation of cell division (Love et al., 2009) and inhibition of cellular division (Edwards and Miller, 1972; Lee and LaRue, 1992; Heidstra et al., 1997; Dubois et al., 2013; Luo et al., 2013). In *Arabidopsis*, ethylene has been associated with reduced petal and leaf size in mutants with constitutive ethylene signaling (Kieber et al., 1993; Roman and Ecker, 1995; Luo et al., 2013) and under water limiting conditions due to the activity of ERF6 through its control of GA2-OXIDASE (Dubois et al., 2013). In the transcriptomic analysis of *dwarfy* leaves we do not see evidence of either *ERF* or *GA2-OXIDASE* genes accumulating at altered abundances. Rather, within the group of genes regulated in *dwarfy* leaves, we observed the repression of a large class of cell cycle genes including *CYCLIN-DEPENDENT KINASE1* (*CDK1*), *CYCLIN A*, and *CYCLIN B1* (Table 2). In eukaryotic cells, CYCLIN A initiates the cellular transition from G2 to prophase after which CYCLIN B1 enters the nucleus and, together with CDK1, induces mitosis by phosphorylation and activation of enzymes regulating chromatin condensation, nuclear membrane breakdown and mitosis-specific microtubule and microfilament re-orientation (Nigg, 2001; Smits and Medema, 2001; Gavet and Pines, 2010; Suryadinata et al., 2010; Rattani et al., 2014). As this whole suite of proteins is necessary for cellular division, repression of their transcription in the leaves of *dwarfy*, as compared to wild-type leaves, is likely the key pathway by which leaf size is being affected. These results are reminiscent of earlier observations that ethylene in *Pisum sativum* stopped cell division prior to entry into prophase (Apelbaum and Burg, 1972).

The leaf drop date of natural grown-year old dwarfy and wild-type plants was assessed as increased ethylene levels have been correlated to early leaf senescence (Breeze et al., 2011; Koyama et al., 2013). Leaf yellowing, considered to be the first visible senescent event (Quirino et al., 2000) was present in only the dwarfy basal leaves in October (Figure 6C), while in the wild-type, senescence related-color changes were prevalent in November basal leaves (Figure 6D) by which time dwarfy basal leaves had dehisced. Buchanan-Wollaston et al. (2003) noted that plants exposed to exogenous ethylene do exhibit premature senescence with the older leaves yellowing first; similar to the results here-in. November dwarfy and wild-type plants both had green apical leaves suggesting the onset of senescence and leaf dehiscence in all but the apical leaves in the dwarfy mutant were altered.

Our results support the hypothesis that there are two different developmental programs regulating tissue size in the *dwarfy* mutant. In the stem, we observe alterations to ethylene response factors and an inhibition of auxin homeostasis genes suggesting that ethylene inhibits stem elongation as previously observed in model organisms such as *Arabidopsis* (Guzman and Ecker, 1990), poplar (Junghans et al., 2004; Love et al., 2009; Vahala et al., 2013), tobacco (Romano et al., 1993), and tomato (Huang and Lin, 2003) through its influence on the auxin pathway. In the leaves, we find that the leaves of *dwarfy* produce fewer cells and are, thereby, smaller. This phenotype is likely tied to the differential expression of the protein group responsible for the induction of mitosis. Altogether, our study of the *dwarfy* mutant poplar has given insight into the genetics underpinning ethylene-induced dwarfism.

### Conflict of interest statement

The authors declare that the research was conducted in the absence of any commercial or financial relationships that could be construed as a potential conflict of interest.

## Supplementary material

Table S1 **Primers used in this study**.

Table S2 **Genes differentially regulated in the stems of *dwarfy* as compared to wildtype stems**.

Table S3 **Genes differentially regulated in the leaves of *dwarfy* as compared to wild-type leaves**.
